# Unraveling the Role of Tumor-Infiltrating Immune Cells in Head and Neck Squamous Cell Carcinoma: Implications for Antitumor Immune Responses and Immunotherapy

**DOI:** 10.3390/ijms26136337

**Published:** 2025-06-30

**Authors:** Paula Constanza Arriola Benítez, Mariel Fusco, Ricardo Amorin, Carlos Rafael Picón, Flavia Piccioni, Lucia Victoria, Manglio Miguel Rizzo, Mariana Malvicini

**Affiliations:** 1 Cancer Immunobiology Laboratory, Facultad de Ciencias Biomédicas, Instituto de Investigaciones en Medicina Traslacional (IIMT), CONICET-Universidad Austral, Av. Presidente Perón 1500, Derqui-Pilar B1629ODT, Buenos Aires, Argentina; carriola-conicet@austral.edu.ar (P.C.A.B.); mfusco-conicet@austral.edu.ar (M.F.); fpiccioni@austral.edu.ar (F.P.); 2 Department of Clinical Oncology, Hospital Universitario Austral, Av. Presidente Perón 1500, Derqui-Pilar B1629ODT, Buenos Aires, Argentina; ramorin@cas.austral.edu.ar (R.A.); cpicon@cas.austral.edu.ar (C.R.P.);

**Keywords:** head and neck cancer, tumor microenvironment, immune checkpoint inhibitors, immune evasion

## Abstract

Head and neck squamous cell carcinoma (HNSCC) is a highly heterogeneous malignancy characterized by a complex tumor microenvironment (TME) that plays a critical role in disease progression and therapeutic resistance. Tumor-infiltrating immune cells, including T lymphocytes, macrophages, dendritic cells, and myeloid-derived suppressor cells, exhibit dual functions, either promoting or suppressing tumor growth depending on their phenotype and interactions within the TME. The presence of immune evasion mechanisms, such as the loss of human leukocyte antigen (*HLA*) expression, upregulation of immune checkpoint molecules, and metabolic reprogramming (hypoxia-induced glycolysis and lactate accumulation), further contributes to immune suppression and poor treatment responses. While immune checkpoint inhibitors (ICIs) have revolutionized the treatment of recurrent/metastatic HNSCC, response rates remain highly variable, underscoring the need for biomarker-driven patient selection and combinatorial therapeutic strategies. This review provides a comprehensive analysis of the role of immune cells in the TME of HNSCC, discusses the mechanisms underlying immune escape, and explores emerging immunotherapeutic and epigenetic-targeting approaches aimed at enhancing antitumor immune responses and improving clinical outcomes.

## 1. Introduction

HNSCC is a heterogeneous group of malignant tumors that originate from the mucosal epithelium of the oral cavity, oropharynx, larynx, hypopharynx, and nasopharynx, with less frequent involvement of the paranasal sinuses or salivary glands [1,2]. HNSCC is the sixth most common cancer worldwide, with approximately 66,920 new cases and 15,400 deaths reported in the United States in 2023 [1,2]. These malignancies predominantly affect older men and are strongly associated with excessive tobacco and alcohol consumption. However, in recent decades, infection with the human papillomavirus (HPV), particularly HPV-16 and, to a lesser extent, HPV-18, has emerged as a major etiological factor in oropharyngeal HNSCC. HPV-positive tumors generally have a better prognosis and exhibit increased sensitivity to therapies such as chemotherapy and radiotherapy [3,4].

Despite advances in multimodal management, including surgery, chemotherapy, and radiotherapy, more than half of HNSCC patients experience locoregional or distant recurrence after treatment. Cisplatin, the standard radiosensitizing agent, is widely used, but its application is often limited by its toxicity. Recently, the introduction of ICIs, particularly those targeting the programmed cell death protein 1 (PD-1)/programmed cell death ligand 1 (PD-L1) axis, has improved survival in patients with recurrent or metastatic (R/M) HNSCC. However, the role of immunotherapy in the curative setting remains uncertain, and clinical responses vary significantly among patients. This highlights the critical need to better understand the factors that regulate tumor–immune system interactions in HNSCC [5,6].

A key element in this interplay is the role of tumor-infiltrating immune cells within the TME, including T lymphocytes, macrophages, dendritic cells (DCs), and natural killer cells (NK), among others. These cells exhibit dual functions, either promoting or inhibiting tumor growth, depending on their phenotype and the context of the TME. Recent studies have shown that the composition, spatial distribution, and functional states of tumor-infiltrating immune cells within the immune microenvironment of HNSCC are closely associated with treatment responses and clinical outcomes. However, the precise mechanisms underlying these interactions remain poorly understood [7,8].

## 2. Immune Landscape in HNSCC

The TME is composed of neoplastic cancer cells surrounded by non-malignant cells embedded in an extracellular matrix, establishing a complex network. Immune infiltration, which includes both innate and adaptive immune cells, is a major component of the TME and plays a fundamental role in tumor progression and the development of an effective antitumor immune response. HNSCCs have been described to exhibit differences in the degree of immune infiltration, which includes macrophages, neutrophils, DCs, NK cells, T and B lymphocytes, and the chemokines and factors that they secrete. Furthermore, the presence of certain mutations, such as the tumor protein 53 (TP53) mutation, can influence the phenotype and abundance of tumor-infiltrating immune cells [9]. In this sense, it has been reported that the presence of an aneuploid-immune paradox encompasses somatic copy-number alteration in HNSCC HPV-negative patients, contributing to an immune hot-to-cold switch during tumorigenesis [10]. In addition to the presence of immune cells, the tumor stroma can influence the growth and progression of HNSCCs. Tumor-associated fibroblasts (CAFs) are abundant in the HNSCC TME and their heterogeneity, plasticity, and dual behavior has been reported in many tumors [11]. CAFs interact with DCs and NK cells but also orchestrate tumor angiogenesis, interacting with endothelial cells (ECs), another component of the TME [11]. Recently, an immunostimulatory CAFs population was observed in high-dimensional single-cell RNA sequencing from advanced HNSCC patients [12]. In this work, the authors reported a subpopulation which is able to reduce the exhaustion of CD8+ cells promoted by the transforming growth factor beta (TGFβ) and increase the NK resident cells and the T cell cytotoxicity. On the other hand, a spatial transcriptomic analysis reported recently identified a phenotype of CAFs expressing Galectin 9 (Gal9) that are involved in CD8+ T lymphocytes’ infiltration and function. CAFs’ Gal9+ promotes CD8+ cell dysfunction and decreases their abundance in HNSCC tumors [13]. ECs can also promote tumor progression. In order to sustain tumor growth, ECs are recruited and induced to generate new vessels through the secretion of vascular endothelial growth factor (VEGF), which is currently the main target of anti-angiogenic therapy [14]. HNSCCs also show, as a part of the TME, intratumoral lymphatic vessels that could be involved in the formation of pre-metastatic niches (PMNs) [15]. More recently, it has been observed that the PMN presents an immunosuppressive environment accompanied by the presence of an exhausted phenotype of CD4+ T cells [16]. In addition to the contribution of stromal cells, tumors often deploy a variety of strategies to subvert the immune response, favoring an immunosuppressive milieu that supports their growth, progression, and invasion [17,18]. Therefore, a deeper understanding of the crosstalk between tumors and immune cells, as well as the mechanisms used by tumors to manipulate and evade immune surveillance within the TME, is essential for improving immunotherapies, identifying predictive biomarkers, and discovering new therapeutic targets or combination strategies.

One of the key immune components of the HNSCC TME is macrophages. These innate immune cells are known for their remarkable phenotypic plasticity, heterogeneity, and functional diversity, enabling them to participate in a wide range of processes. Tumor-associated macrophages (TAMs) are a prominent component of the tumor stroma, with phenotypes ranging from M1-macrophages (antitumor, pro-inflammatory), which promote cytotoxic responses against tumors, to M2-macrophages (pro-tumor, immunosuppressive), which support tumor progression and suppress immune activity [19,20]. In the HNSCC milieu, macrophages are attracted and act mainly in an immunosuppressive manner. Several clinical studies revealed that total TAMs (CD68+) and particularly M2-macrophages (CD163+) are abundant in the TME and are negatively correlated with prognosis in HNSCC patients [21,22,23].

Neutrophils are a crucial component of the immune system’s first line of defense against pathogens, responding to various inflammatory signals, including those in the TME. Similarly to macrophages, tumor associated-neutrophils (TANs) can exhibit opposing functions depending on the context, with either antitumoral or protumoral effects through direct and indirect mechanisms [24]. In recent years, special attention has been given to neutrophil extracellular traps (NETs), web-like structures composed of DNA, histones, and granule enzymes [25]. NETs may play a role in tumor initiation, progression, and metastasis [26,27]. Remarkably, advanced oral squamous cell carcinoma (OSCC) patients (stages III/IV) exhibited augmented levels of plasma NETs compared to those in early stages of the disease (I/II) and healthy age- and sex-matched controls [28]. Furthermore, NETs contribute to a hypercoagulable state in advanced-stage patients, which may exacerbate the risk of complications [28].

NK cells are large, granular lymphoid cells that are a fundamental component of the innate immune system and play a pivotal role in the surveillance of virus-infected cells and cancer cells. NK cells are mainly produced in the bone marrow and, once mature, enter the bloodstream, migrate and settle in peripheral tissues [29]. NK cells are complex and unique. They become activated upon recognizing malignant cells primarily through the loss of MHC-I expression on the cell surface. Activated NK cells trigger cytotoxicity and produce inflammatory cytokines -interleukin (IL)-2, IL-12, IL-15, interferon gamma (IFN-γ), and tumor necrosis factor alpha (TNF-α)- which help recruit other immune cells and initiate the adaptive immune response to eliminate tumors [30].

Considering their potent immunosuppressive effects, myeloid-derived suppressor cells (MDSCs) are a key component of the TME in HNSCC. MDSCs are immature myeloid cells characterized by their heterogeneity and are classified into polymorphonuclear (PMN)-MDSCs and monocytic (M)-MDSCs based on their morphology and phenotype [31]. MDSCs experience a substantial increase during tumor progression and play a crucial role in facilitating tumor immune escape, metastasis, and treatment resistance via multiple mechanisms. In the TME, MDSCs suppress T cell function by not only increasing the expression of inhibitory immune checkpoint molecules expression, such as PD-L1, and depleting amino acids essentials for T cell function, but also producing adenosines, reactive oxygen (ROS) and nitrogen species (RNS) and impairing T-cell trafficking to tumors [31,32].

Despite the presence of a robust innate immune system within the TME, adaptive immune systems play crucial roles in immune-mediated tumor elimination. Most adaptive responses against tumors are mediated by CD4+ and CD8+ lymphocytes, which recognize and destroy neoplastic cells [33]. DCs act as sentinels, constantly patrolling the human body. When they encounter tumor antigens, these are phagocytized and presented on their surface via the major histocompatibility complex (MHC)-II. These antigens are identified by T cells and naïve T cells in secondary lymph nodes and undergo maturation into effector cells. Naïve CD4+ T cells can differentiate into T helper (Th) cells and T regulatory cells (Tregs). Among the Th subsets, IFN-γ produced by Th1 cells exerts a critical role in tumor elimination. CD4+ T cells are necessary for the proliferation and differentiation of CD8+ T cells, the generation of memory CD8+ T cells, and their infiltration into the tumor [34,35]. Cytotoxic CD8+ T cells (CTLs) are the most potent effectors in the antitumor immune response [36]. CTLs are an integral component of the TME in HNSCC. Recent studies have shown that augmented CD8+ T-cell infiltration is significantly correlated with improved prognosis in HNSCC patients. In contrast, impaired T-cell counts have been reported in both the tumor and circulating T lymphocytes of HNSCC patients [37]. Oliveira et al. analyzed T cell activation and dynamics in responders and non-responders to neoadjuvant pembrolizumab in HNSCC patients, finding that tumors responding to pembrolizumab contained a population of tumor-infiltrating CD8+ T cells expressing genes associated with T cell exhaustion, tissue residence, and cytotoxic potential [38].

Lastly, other components of the TME are B lymphocytes, adaptive immune cells responsible for secreting antibodies and antigen presentation. There are different subtypes of B cells, which can engage in both tumor-promoting and antitumoral activity, via antibody and cytokine production, co-stimulation, and antigen presentation. Recent studies demonstrated that activated and memory B cells are enriched in the TME of HNSCC. Furthermore, other studies have identified several subsets of regulatory B cells (Bregs) in the tumor-draining lymph nodes, associated with favorable outcomes [39,40,41].

## 3. Mechanisms of Immune Evasion as a Challenge to Therapeutic Effectiveness

The success of cancer immunotherapy has demonstrated that immune cells, particularly T cells, can be harnessed to eliminate tumor cells [42]. Both subtypes of HNSCC, associated with the HPV (HPV-positive) and the HPV-negative tumors, have been shown to exhibit the highest levels of immune infiltration. Conversely, most patients do not respond to treatment, likely due to primary and acquired resistance mechanisms involving immunosuppressive effectors and cells which promote tumor growth and are also present within the TME.

Several mechanisms of immune evasion have been described, including (i) the altered expression of HLA class I to avoid antigen detection and presentation; (ii) changes in the number and function of immune cell populations; (iii) regulation of cell-to-cell contact points, such as immune checkpoints; (iv) secretion of VEGF, TGFβ, IL-6, IL-10, and GM-CSF cytokines, all of which support the escape from the recognition and elimination of transformed cells. In addition to the upregulation of immune checkpoints, like PD-L1, cytotoxic T lymphocyte antigen 4 (CTLA-4), lymphocyte-activation gene 3 (LAG-3), T cell immunoglobulin and ITIM domain (TIGIT), and T cell immunoglobulin and mucin domain-containing protein 3 (TIM-3), other characteristics of the TME, including hypoxia, acidic pH, lactate accumulation, anomalous vasculature, and high intratumoral pressure, may influence the trafficking, infiltration and function of immune cells.

Moreover, the integration of immunological analyses with genomics has provided further insight into tumor behavior in recent years. Understanding these mechanisms is crucial for developing effective immunotherapeutic approaches [43].

### 3.1. Loss of Human Leukocyte Antigen and Impairment of Antigen Presentation

T lymphocytes must recognize overexpressed tumor antigens or neoantigens derived from mutations to mediate cancer cell killing. The antigens are first processed and then presented through the antigen presentation pathway by the major histocompatibility complex in humans, known as human leukocyte antigens (*HLAs*) and codified by a family of highly polymorphic genes [44]. Therefore, tumors can evolve to reduce HLA expression and evade immune surveillance. This phenomenon has also been described in HNSCC. To reduce HLA expression, cancer cells can induce the loss of either the maternal or paternal copy of an *HLA class I* genes, a process referred to as a loss of *HLA* heterozygosity (*HLA* LOH) or promote the loss of one of the *HLA* alleles. Both mechanisms reduce the variety of tumor antigens presented to T cells, thereby decreasing their visibility to the immune system. Recently, a study by Morris [45] analyzed the clinical and RNA-sequencing data from The Cancer Genome Atlas (TCGA) of 522 HNSCC patients, focusing on their infiltrating immune populations and the expression of *HLA class I* genes based on previous reports from Gong and Karchin and Li et al. [46,47]. The study identified that *HLA* LOH is the primary mechanism of *HLA* alteration, present in more than 50% of patients. Notably, LOH was slightly higher in HNSCC than in other cancer types analyzed in the TCGA. Additionally, *HLA* LOH was more prevalent in advanced stages of HNSCC than in early stages. Although most tumors with HLA LOH exhibited the loss of more than one *HLA class I* genes, *HLA* LOH was not associated with their expression, indicating that this occurrence had no clear impact at the transcript level. The deconvolution of RNA-seq data showed that tumor infiltrating immune cells were fewer in HNSCC with *HLA* LOH. Particularly, in HPV-negative HNSCC, this reduction was significant for infiltrating CD8+ cytotoxic T cells and naïve B cells [45].

Finally, this report along with other analyses of HNSCC, found no association between *HLA* status and clinical response to immunotherapy [48]. Additionally, studies in other solid tumors suggested that *HLA* loss or LOH may not be a determinant of survival outcomes but could serve as one of the immune escape mechanisms used by tumors.

Regarding epigenetic modifications, it has been reported that the hypermethylation of CpG islands in *MHC-I* promoter regions leads to reduced expression, and approximately 66% of HNSCC samples exhibit hypermethylation [49]. Another mechanism involves the epidermal growth factor receptor (EGFR) signaling pathway. Nearly 80% of HNSCCs overexpress EGFR, which influences *MHC-I* expression by modulating a class II major histocompatibility complex transactivator (CIITA), a key transcriptional regulator [50].

As another mechanism to elude immune recognition, HNSCC cells can downregulate or mutate other components of antigen presentation machinery [51]. Previous analyses have reported that mutations in genes beta 2 microglobulin (B2M) and transforming growth factor receptor 2 (TGFBR2) may contribute to impaired immune surveillance in HNSCC [52]. Additionally, up to 20% of TCGA HNSCC tumors exhibit mutations in genes from proteasome-associated proteins involved in antigen presentation, such as calnexin, latent membrane protein (LMP2, LMP7, LMP10), and calreticulin, as well as lower levels of tapasins (TAP1 and TAP2), and endoplasmic reticulum protein 57 (ERp57) [53].

### 3.2. Disruption of Immune Cell Infiltration, Secretion of Immunosuppressive Factors, and Recruitment of Suppressor Cells

HNSCC tumors can prevent immune cell infiltration by disrupting chemoattractant pathways, thereby hindering the recruitment of immune cells to the tumor site. Both the absence of immune cells and the presence of functionally impaired effector cells, such as NK cells or cytotoxic CD8+ cells, have been reported, as well as the presence of immunosuppressive cells, including TAMs, MDSCs, and Tregs and Bregs [54].

#### 3.2.1. Innate Immune Cells

##### Dendritic Cells

DCs are often recruited to the TME, where they are manipulated into an immunosuppressive state by HNSCC-derived cytokines, such as IL-10, VEGF, and TGFβ, which prevent the proper activation of T cells. Overexpressed immunosuppressive cytokines convert DCs into tolerogenic DCs, which induce Tregs instead of cytotoxic T cells. Additionally, the secretion of VEGF and IL-10 causes the downregulation of MHC-II and CD80/CD86, and immature DCs accumulate in the TME, impairing T cell activation [55].

HNSCC cells also recruit immunosuppressive plasmacytoid DCs (pDCs) via the secretion of C-X-C motif chemokine ligand (CXCL)12 and CXCL14; pDCs secrete IL-10 further recruit more Tregs, dampening the immune attack even more [56,57]. Then, DCs in HNSCC tumors become dysfunctional, failing to activate cytotoxic T cells and promoting an immunosuppressive environment.

##### Tumor-Associated Macrophages

In the HNSCC milieu, chemokines such as chemokine C-C motif ligand (CCL)3, CCL4, CCL5, and monocyte colony-stimulating factor (MCSF), as well as VEGF, IL-10, IL-4, and IL-13, are secreted to attract macrophages into the tumor and skew them towards the M2 phenotype. Like tolerogenic DCs, secreted factors like TGFβ, IL-10, and Arginase 1 (ARG 1), which depletes arginine—a key nutrient for T cell activation—and VEGF secreted by M2-macrophages inhibit cytotoxic T cell and NK cell function while promoting the expansion of Tregs. M2-macrophages also express immune-modulating molecules such as PD-L1, PD-L2, and CTLA-4, which dampen T cell responses by blocking key co-stimulatory signals for T cell activation. HNSCC tumors reprogram macrophages into M2-like cells, which actively suppress T cells and NK cells while promoting tumor survival [58].

##### Neutrophils

In recent years, the role of neutrophils in cancer has become better understood. In fact, an increase in both circulating and tumor-infiltrating neutrophils has been observed in some types of solid tumors. In particular, higher neutrophil infiltration and elevated neutrophil-to-lymphocyte ratios (NLR) have been associated with poor outcomes in patients with HNSCC [59,60]. Although peripheral neutrophils have been identified as biomarkers of poor clinical prognosis in several cancer types, the predictive value of TANs remains under investigation. Some reports suggest that both TANs and circulating neutrophils may influence the efficacy of treatments [61].

HNSCCs attract neutrophils via IL-8 secretion, and TGFβ induces neutrophils to shift into an N2 phenotype, which suppresses immune responses. TANs secrete ARG 1 and IL-10, blocking T cell responses. In addition, N2 neutrophils induce CD8+ T cell apoptosis through TNF-α and nitric oxide (NO) pathways. Another study reported that CXCL5 expression is significantly upregulated in laryngeal squamous cell carcinoma (LSCC), and elevated CXCL5 expression correlates strongly with increased intratumoral neutrophil infiltration. When compared to peripheral blood neutrophils, TANs demonstrated a pronounced inhibitory effect on T-cell proliferation and reduced the secretion of IFN-γ and TNF-α. Moreover, the study suggests that excessive neutrophil infiltration is linked to more advanced clinical stages of LSCC (T3 or T4, III or IV, and N1 or N2), potentially contributing to disease progression [62]. Thus, in HNSCC, neutrophils adopt a suppressive characteristic instead of an inflammatory one, helping tumors evade the immune system [63].

##### Myeloid-Derived Suppressor Cells

MDSCs help regulate inflammation and prevent excessive immune activation. They are recruited through granulocyte/monocyte-colony stimulating factor (GM-CSF), IL-6, VEGF, Prostagladin E2 (PGE2), Indolamine-2,3-dioxygenase (IDO), and other HNSCCs-derived signals and factors. MDSCs inhibit T cell function by depleting essential nutrients (e.g., arginine and tryptophan) and producing NO, ROS, and peroxynitrite. NO and ROS production causes oxidative stress and peroxynitrite damages T cells and alters T cell receptor (TCR) signaling, making T cells unresponsive. Infiltrating MDSCs also starve T lymphocytes and NK cells by depleting arginine and tryptophan, inducing cell dysfunction and anergy. Then, MDSCs can block immune responses by suppressing the availability of metabolites, starving immune cells of essential nutrients, and producing suppressive molecules. In OSCC tumors, the number of MDSCs is increased and positively related to the T stage, pathological grade, lymph node metastasis and poor prognosis [64,65].

#### 3.2.2. Adaptive Immune Cells

##### Regulatory T Cells

HNSCCs can recruit Tregs through chemokines CCL2, CCL28, and CXCL12, which interact with Tregs receptors CCR4, CCR10, and CXCR4, respectively. HNSCC also secretes cytokines TGFβ, IL-10, and IL-35, which promote the differentiation of naïve CD4+ T cells into Tregs. Once in the TME, these cytokines, along with the presence of adenosine (produced by CD39/CD73) further enhance Tregs differentiation and activation. At this stage, activated Tregs suppress cytotoxic immune responses by inhibiting T cells, NK cells, and DCs through IL-10 and TGFβ. Tregs can also directly kill immune cells by secreting granzyme B and perforin, or by triggering Fas-mediated apoptosis in CD8+ T cells. Thus, Tregs in the TME actively suppress antitumor immune responses, allowing the tumor to evade immune detection.

##### Regulatory B Cells

Bregs have also been identified as mediators of immunosuppression in the HNSCC TME [66]. Among other mechanisms, Bregs inhibit T cell function through the production of adenosine [67]. Adenosine-producing Bregs were found both in blood and in tumor samples of HNSCC patients, and they were able to inhibit the activity of Bruton tirosin kinase (BTK) and the influx of intracellular calcium, affecting the activity of effector B cells, which are associated with antitumor functions and improved progression-free survival (PFS) in HNSCCs [68].

#### 3.2.3. Non-Immune Cells

CAFs create a physical and biochemical barrier against immune infiltration. HNSCC recruits CAFs via FAP, PDGFA, and TGFβ. In the TME, CAFs secrete TGFβ, IL-6, VEGF, and CXCL12, which enhance immune suppression and promote Treg activation. CAFs also upregulate PD-L1/PD-L2, leading to reduced T cell activity [69].

#### 3.2.4. Secretion of Immunosuppressive Factors and Recruitment of Suppressor Cells

In addition to soluble factors or chemokines derived from the tumor that promote tumor infiltration of suppressor cells, genomic aberrations can profoundly influence immune access in tumors. First, *HLA* LOH has been linked to a reduction in CD8+ T cells, Tregs, and B cells in other tumors [55,70,71]. Furthermore, TP53—one of the most frequently mutated genes in HNSCC—is associated with lower levels of infiltrating B cells, CD8+ T cells, and NK cells. Another critical gene implicated in cancer progression is *CUB* and *Sushi Multiple Domains 1* (*CSMD1*), a tumor suppressor gene which is involved in regulatory cellular functions and is lost in approximately 40% of HNSCC tumors. It has been observed that reduced or absent *CSMD1* correlates with decreased T cell infiltration. Finally, a widely studied pathway involving the phosphoinositide 3-kinase (PI3K), which appears frequently mutated in HNSCC, plays a pivotal role in cancer progression [72]. Additionally, a downstream target of this pathway is *phosphatase and tensin homolog* (*PTEN*). Loss of *PTEN* leads to increased expression of chemokines CCL2 and VEGF, which, as we described above, can impede T cell infiltration [73].

### 3.3. Expression of Immune Checkpoint Molecules

Tumor cells in HNSCC often upregulate immune checkpoint molecules, such as PD-L1, which bind to their receptors on T cells, leading to T cell exhaustion and reduced antitumor activity. This mechanism allows tumor cells to escape immune surveillance and continue to proliferate.

The widely described PD-1/PD-L1 axis participates in modulating immune cells and other stromal cells and has been proposed as an additional potential mechanism for immunosuppression, facilitating immune evasion and resistance [74]. In addition to the ability to bind to PD-1, PD-L1 can bind to CD80 (B7-H1), which provides inhibitory signals in T cells. Particularly, in HNSCC, the enhanced expression of PD-L1 in the TME, not only on tumor cells but also in M2-macrophages, was associated with decreased levels of effector CD4+ and CD8+ cells [75].

CTLA-4 shares the ligand with the stimulatory molecule CD28; however, when CTLA-4 binds to CD80 or CD86, it delivers inhibitory signals, suppressing the activation of T cells. A high expression of CTLA-4 was found in human HNSCC samples, associated with a lower presence of CD8+ T cells and poor prognosis. Additionally, the expression of CTLA-4 was also associated with higher populations of circulating Tregs and with MDSC-related chemokines and MDSC-gene expression profiles [76].

In addition to the strategies aimed at blocking PD-1/PD-L1 and CTLA-4, other immune checkpoint proteins may influence the development of an effective antitumor immune response which are being extensively studied.

TIGIT is a receptor expressed on activated T and NK cells [77], which binds to CD155 to induce immunosuppression. An enhanced expression of TIGIT on tumor-infiltrating CD8+ and CD4+ T cells in HNSCC patients was correlated with CD8+ functional exhaustion and impaired activation and proliferation as well as with the expression with other immune checkpoint molecules such as PD-1 [78].

TIM-3 is expressed in Tregs population and it is associated with Th1 lymphocytes suppression. Additionally, it cooperates with TIGIT to induce T cell exhaustion [79]. It has been reported that circulating TIM-3+ TIGIT+ Tregs from HNSCC patients showed a high capacity for impeding T-cell proliferation by secreting granzime B and suppressive cytokines such as IL-10.

V-domain Ig suppressor of T cell activation (VISTA) has been referred to as a PD-1 homolog expressed in APC, naïve T cells, and Tregs [80]. In HNSCC, the expression of VISTA has been associated with MDSCs, particularly in OSCC. Additionally, it has been reported that through the interaction with its ligand VSIG-3 (V-Set and immunoglobulin domain containing 3), VISTA promotes Treg maturation and inhibits the proliferation of T effector cells. In this context, high expression of VISTA, together with low expression of CD8, has been reported to correlate with significantly poorer overall survival (OS) in OSCC patients [81].

LAG-3 is mainly expressed on effector CD4+ and CD8+ T cells, NK cells, B lymphocytes, DCs, and Tregs and is also considered as the third immune check point, since LAG-3 binds to MHC-II [82], leading to immune cell suppression. In HNSCC, an overexpression of LAG-3 has been reported on the TME. Moreover, this upregulation is regarded as a marker of T cell exhaustion, since it has been correlated with inhibition of CD8+ T lymphocytes and increased levels of Tregs. In addition, the co-expression of LAG-3 and PD-1 has also been reported on tumor-infiltrating CD8+ cells [83].

### 3.4. Metabolic Changes in the Tumor Microenvironment

The TME in HNSCC undergoes metabolic and structural changes, such as hypoxia, which can influence the behavior of both tumor and immune cells. These changes can create an immunosuppressive environment that supports tumor progression and resistance to therapy.

For instance, the metabolic reprogramming of cancer cells, characterized by the Warburg effect, has been considered a driver of tumor progression [84]. This adaptation provides tumors with an advantage in competing for survival in hostile hypoxic and acidic TME. In the competition for nutrients, especially glucose, the Warburg effect enhances aerobic glycolysis in tumor cells under hypoxic conditions, while immune cells mainly rely on oxidative phosphorylation for energy. As a result, cancer cells continue to consume glucose, limiting its availability for T cells and directly reducing their effector function [84].

Additionally, tumor cells under Warburg effect secrete a large amount of lactic acid, which among other effects, induce M2 polarization of TAMs through histones lactylation [85].

In this scenario, the proteins involved in lactate metabolism and transport play a critical role in tumor reprogramming. The association between lactate dehydrogenase family genes expression and their alteration in HNSCC has been recently investigated [86]. Xu et al. have observed that high expression of LDH isoform B (LDHB) in tumor samples of patients from TCGA database was associated with diminished immune cell infiltration and cytotoxic T cells dysfunction, while low LDHB expression was associated with reduced tumor cell proliferation and ATP production, improved immune cell infiltration, and better response to immunotherapy.

In another study by Du et al., a metabolism-related gene prognostic index (MRGPI) connecting metabolic characteristics with antitumor immune response has been reported in HNSCC. Between the identified metabolism-related genes associated with the prognosis of HNSCC patients are 1-acylglycerol-3-phosphate O-acyltransferase 4 (AGPAT4); amylase alpha 2B (AMY2B); acyl-CoA dehydrogenase long-chain (ACADL); creatine kinase, muscle (CKM); and adenosine deaminase (ADA), which are related to energy and purine, phospholipids, and mitochondrial beta oxidation of fatty acid, among other metabolic proteins [87]. The authors observed that patients with the low-MRGPI exhibited low metabolic activities, active antitumor immune response and better OS, while patients with high-MRGPI group showed high metabolic activities and a low antitumor immune response.

## 4. Strategies to Counteract Immune Escape Mechanisms in HNSCC Patients

**Figure 1:**
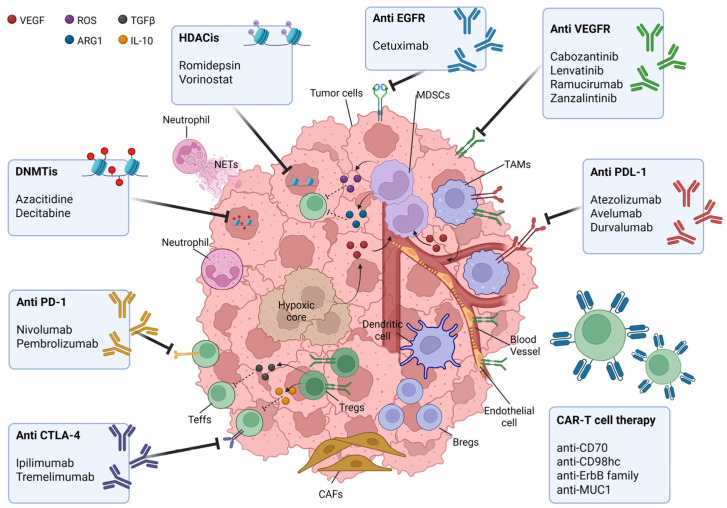
Current therapeutic strategies to overcome the immunosuppressive HNSCC TME. HNSCC features a TME where tumor-infiltrating immune cells mediate antitumor immunity and immune evasion. HNSCC employs Tregs and MDSCs to impede the activity of effector T cells (Teffs). Tregs secrete IL-10 and TGF β, suppressing Teff cytotoxicity. MDSCs, driven by IL-6 and GM-CSF, produce arginase-1 and ROS, impairing T cell function. Additionally, the HNSCC TME fosters evasion through hypoxia, acidosis, and stromal barriers, while M2-polarized TAMs secrete VEGF, boosting MDSC recruitment. NETs shield tumors, and VEGF-driven angiogenesis exacerbates immunosuppression. PD-L1 overexpression (50–70% of HNSCC) on tumor cells and immunosuppressive TME cells engages PD-1, inducing Teffs anergy. ICIs as anti-PD-1/PD-L1 therapies have improved clinical outcomes, and alternative checkpoints (LAG-3, TIM-3, TIGIT, CTLA-4) have also been approved for HNSCC; however, response rates are over 15–20% in R/M cases. This multi-checkpoint network challenges single-agent therapies, urging combined blockade strategies. Emerging therapies include CAR-T cells, epigenetic modulators such as DNMTis and HDACis, bi-specific antibodies targeting angiogenesis and antibody–drug conjugates show potential to bypass immunosuppression in tumor tissues and simultaneously enhance the antitumor activity of immune cells.

HNSCC employs multiple strategies to manipulate tumor-infiltrating immune cells, enabling immune escape, resistance to immune attacks, suppression of immune responses, and recruitment of immunosuppressive cells. While conventional treatments provide benefits for patients in the early stages of the disease, resistance to these therapies remains a significant challenge in advanced HNSCC [88]. Addressing the complex immune evasion network through multifaceted therapeutic approaches holds promise for enhancing treatment efficacy and improving patient outcomes. ICIs represent a groundbreaking advancement in cancer therapy, demonstrating encouraging results in HNSCC. However, their role in curative setting remains uncertain. Recent advancements in chimeric antigen receptor (CAR)-T cell engineering, combination therapies, and strategies to modulate the TME are paving the way for more effective and sustainable immunotherapeutic approaches in HNSCC. Additionally, targeting epigenetic alterations offers a promising frontier in HNSCC treatment, with the potential to improve patients’ outcomes and long-term survival (Figure 1).

### 4.1. Chemotherapy

In the early stages (I and II) of HNSCC, the treatment is predominantly surgical, often utilizing advanced, minimally invasive techniques [89]. On the other hand, for patients with locoregionally advanced HNSCC (LA-HNSCC) or those presenting with positive margins after surgery, the treatment protocol generally includes a combination of radiotherapy (RT) and concurrent cisplatin-based chemotherapy (CRT), in addition to surgery [89]. A site-specific meta-analysis published in 2011 demonstrated consistent benefits from adding chemotherapy to locoregional treatment (LRT), with a 13% relative reduction in death (pooled HR 0.87, 95% CI 0.84–0.91) [90]. This landmark analysis established CRT as the standard of care for most patients with LA-HNSCC.

Based on these results, an updated meta-analysis published in 2021 reaffirmed the advantages of concurrent CRT, showing a 6.5% survival benefit at five years and 3.6% at ten years. Furthermore, this approach was found to be superior in improving OS by 6.2%, event-free survival (EFS) by 3.7%, and reducing locoregional failure (LRF) by 5.8% at five years, when compared to induction chemotherapy, including taxane-containing triplet regimens [91].

In cases where chemotherapy may not be a suitable option, Cetuximab, an EGFR inhibitor, can be considered as an alternative. While Cetuximab, when added to cisplatin and radiotherapy, increased toxicity without significantly improving survival, it remains a viable option for selected patients with HPV-negative LA-HNSCC undergoing treatment with systemic therapy plus concurrent radiotherapy [92]. Despite these progresses, around 15–40% of patients with locally advanced HNSCC are at risk of local recurrence, and even distant metastasis, with a 5-year OS rate of only 50% [93]. This highlights the urgent need for research into strategies aimed at improving the outcomes of current treatments for locoregionally advanced HNSCC.

In the context of R/M HNSCC, clinical trials have traditionally relied on a chemotherapy regimen consisting of platinum-based agents (cisplatin or carboplatin) in combination with 5-fluorouracil (5-FU). While this treatment has been effective, it is also associated with significant adverse effects, including diarrhea and mucositis [94]. High-dose cisplatin (100 mg/m^2^ every three weeks) or carboplatin combined with 5-FU have been tested in randomized controlled trials and have demonstrated improvements in disease-free survival (DFS) and OS in patients with stage III/IV oropharyngeal squamous cell carcinoma (OPSCC) [95].

Both cisplatin monotherapy and combination therapy with platinum and 5-FU have shown to provide similar benefits, with high-dose cisplatin continuing to be the preferred radiosensitizer for CRT [96]. An alternative strategy currently being explored is the use of weekly high-dose cisplatin, which offer more frequent administration while maintaining efficacy [97,98].

For patients in low-income countries, oral metronomic chemotherapy has emerged as a more cost-effective treatment option. This approach involves the administration of lower doses of chemotherapy at regular intervals, without interruption, and may be considered as a second-line or platinum-refractory settings. Common drugs in this regimen include celecoxib (200 mg twice daily) and low-dose methotrexate or docetaxel (15 mg/m^2^ weekly) [99,100].

Over the past two decades, immunotherapy has shown significant improvements in the treatment of advanced HNSCC. It has demonstrated clinical efficacy in patients with unresectable or metastatic HNSCC who are not candidates for surgery or radiation therapy. Currently, the anti-PD-L1 monoclonal antibody pembrolizumab, either as monotherapy or in combination with platinum and 5-FU, is the first-line therapy. The KEYNOTE-048 trial demonstrated that pembrolizumab monotherapy achieved a 17% overall response rate (ORR), while both pembrolizumab with chemotherapy and cetuximab with chemotherapy resulted in a 36% overall response rate [101]. In cases where immunotherapy is not a viable option, the previously established first-line regimen known as EXTREME—comprising platinum, 5-FU, and cetuximab—remains a viable alternative for patients who can tolerate triplet therapy, particularly for those with HPV-negative disease [102]. However, despite these advances, resistance to conventional treatments remains a significant challenge for patients with advanced HNSCC [89].

### 4.2. Immune Checkpoints Inhibitors

ICIs targeting the PD-1/PD-L1, CTLA-4, and other pathways have revolutionized the treatment landscape for R/M HNSCC. While these ICIs share similar mechanisms, they differ in pharmacological characteristics like antibody type, half-life, and antibody-dependent cell-mediated cytotoxicity (ADCC) capacity (Table 1).

#### 4.2.1. PD-1/PDL-1

PD-1 and PD-L1 inhibitors have emerged as transformative therapies for R/M HNSCC since 2013 with the first Phase 1b trial KEYNOTE-012. This trial evaluated pembrolizumab in patients with R/M HNSCC and demonstrated early evidence of efficacy, with an ORR of 18% and durable responses, paving the way for further development of PD-1 inhibitors in HNSCC [103,104].

Pembrolizumab is a humanized immunoglobulin G4 (IgG4) monoclonal antibody designed to target PD-1 receptor, an immune checkpoint expressed on T cells. Unlike IgG1 antibodies, the IgG4 isotype has minimal ADCC and complement-dependent cytotoxicity (CDC) capacity, though it reduces the risk of unintended immune cell destruction. By binding to PD-1, pembrolizumab blocks its interaction with PD-L1 and PD-L2, which are frequently overexpressed on tumor cells and immune cells within the TME. This blockade prevents T-cell suppression, restoring antitumor immune responses and promoting cancer cell elimination [104].

This PD-1 inhibitor has been extensively studied in pivotal trials. The KEYNOTE-048 phase III trial compared pembrolizumab monotherapy, pembrolizumab combined with chemotherapy, and cetuximab combined with chemotherapy in R/M HNSCC. Pembrolizumab monotherapy significantly improved OS in patients with PD-L1 CPS ≥ 20 (HR: 0.61) and CPS ≥ 1 (HR: 0.74). The combination of pembrolizumab with chemotherapy also showed improved OS across all subgroups (HR: 0.71). Based on these results, pembrolizumab received Food and Drug Administration (FDA) approval as a first-line treatment for R/M HNSCC in patients with PD-L1 CPS ≥ 1 (monotherapy) and CPS < 1 (in combination with chemotherapy) [101,105]. In addition, KEYNOTE-689 (NCT03765918) is the first phase III trial to show EFS benefit with neoadjuvant pembrolizumab in resected locally advanced head and neck carcinoma (LAHNC).

In the KEYNOTE-040 phase III trial, pembrolizumab was compared to standard-of-care treatments (methotrexate, docetaxel, or cetuximab) in platinum-refractory R/M HNSCC. The trial reported a median OS of 8.4 months for pembrolizumab vs. 6.9 months for the control group, with fewer grade 3–4 treatment-related adverse events (13% vs. 35%). These findings led to FDA approval of pembrolizumab as a second-line treatment for R/M HNSCC following platinum-based therapy [106].

Nivolumab is a fully human IgG4 monoclonal antibody that binds to PD-1 receptor on T cells. Unlike humanized antibodies, nivolumab’s fully human structure reduces immunogenicity, minimizing the risk of neutralizing antibody formation. Its IgG4 isotype, like pembrolizumab, avoids ADCC and complement activation, preserving immune cells while blocking PD-1-mediated immunosuppression. By preventing PD-1/PD-L1 ligation, nivolumab restores T-cell effector function, enhancing antitumor immunity [107,108]. This inhibitor was evaluated in the CheckMate 141 phase III trial, which aimed at comparing nivolumab (3 mg/kg every 2 weeks) to the investigator’s choice of therapy (methotrexate, docetaxel, or cetuximab) in platinum-refractory R/M HNSCC. Nivolumab demonstrated a response rate of 13.3% compared to 5.8% for the control group, with a 1-year survival rate of 36% vs. 16.6%. The median OS was 7.5 months for nivolumab vs. 5.1 months for the control group (HR: 0.70). Nivolumab also had a favorable safety profile, with fewer grade 3–4 adverse events (13.1% vs. 35.1%). These results led to FDA approval of nivolumab for R/M HNSCC in 2016 [109].

The success of pembrolizumab and nivolumab in phase III trials underscores the importance of PD-1/PD-L1 inhibition in HNSCC. The correlation between PD-L1 expression (CPS) and treatment efficacy highlights the role of biomarker-driven patient selection. Both agents have demonstrated superior survival outcomes and manageable toxicity profiles, establishing them as cornerstone therapies in advanced HNSCC.

Other PD-1/PD-L1 inhibitors are atezolizumab, durvalumab, and avelumab. Atezolizumab was evaluated in the PCD4989g phase Ia trial, which included 32 patients with R/M HNSCC. Results evidenced an ORR of 22%, with a median duration of response of 7.4 months. Notably, 53% of patients had received two or more prior lines of therapy, suggesting activity in heavily pretreated populations [110]. The HAWK study evaluated durvalumab monotherapy, demonstrated an ORR of 16.2% in immunotherapy-naïve patients with R/M HNSCC and PD-L1 tumor expression ≥ 25%. The median OS was 7.1 months, and the 12-month OS rate was 33.6%, indicating moderate efficacy in this patient population [111]. Lastly, avelumab was evaluated in the JAVELIN study. Avelumab showed an ORR of 13.1% in patients with platinum-refractory R/M HNSCC, comparable to other PD-1/PD-L1 inhibitors [112].

However, these results were not reproduced in early stages when immunotherapy was combined with radiotherapy. Large phase III trials (JAVELIN Head and Neck 100 (NCT02952586); KEYNOTE-412 (NCT03040999); GORTEC 2015-01 (REACH) (NCT02999087)) have largely failed to show significant PFS or OS benefits when immunotherapy is added to standard CRT in unselected LAHNC populations. This suggests that concurrent immunotherapy may not synergize effectively with CRT without better patient stratification. PD-L1 expression, tumor mutational burden (TMB), and immune infiltrate are being explored as predictors of response, but no consistent biomarker has emerged for LAHNC with CRT plus immunotherapy.

Emerging evidence suggests that perioperative administration of PD-1 inhibitors, either as neoadjuvant or adjuvant therapy, may offer superior clinical benefits compared to the conventional combination of chemoradiotherapy and immune checkpoint inhibitors. Preliminary results from the KEYNOTE-689 and NIVOPOSTOP trials, as reported by the sponsors in press releases, indicate statistically significant improvements in EFS [113]. These findings, pending publication in peer-reviewed journals, have the potential to redefine current standards of care in this setting.

#### 4.2.2. CTLA-4

CTLA-4 inhibitors, such as tremelimumab and ipilimumab, have been explored in HNSCC, though with less consistent results compared to PD-1/PD-L1 inhibitors. The combination of durvalumab (a PD-L1 inhibitor) and tremelimumab (a CTLA-4 inhibitor) has been evaluated in several trials. The CONDOR phase II trial assessed durvalumab alone or in combination with tremelimumab in patients with PD-L1-low/negative R/M HNSCC. While the combination showed durable responses and a manageable safety profile, it did not significantly improve OS or PFS compared to standard therapies [100].

The EAGLE phase III trial compared durvalumab alone or in combination with tremelimumab to standard-of-care chemotherapy in platinum-refractory or second-line R/M HNSCC. Similarly to the CONDOR trial, the EAGLE trial did not show significant improvements in OS or PFS for the combination therapy. However, the combination demonstrated more durable responses and lower incidence of severe treatment-related adverse events compared to chemotherapy [114].

Ipilimumab plus nivolumab was compared as a first-line treatment in the Checkmate 651. Unselected PD-L 1 patients were randomized to immunotherapy combination vs. platinum-based chemotherapy plus cetuximab. This was another negative study with no differences in OS [115].

Ipilimumab has been studied in combination with cetuximab and intensity-modulated radiation therapy (IMRT) in advanced HNSCC. The NCT01935921 trial reported a 3-year DFS rate of 72% and a 3-year OS rate of 72%, suggesting potential benefits in multimodal therapy [116].

While CTLA-4 inhibitors are shown to be promising in other cancers, their efficacy in HNSCC has been limited. Durvalumab and tremelimumab combinations have demonstrated durable responses and a favorable safety profile but have not significantly improved survival outcomes. Ipilimumab has shown potential in combination with radiotherapy and cetuximab, but further research is needed to define its role. The lack of significant survival benefits with CTLA-4 inhibitors in HNSCC may reflect the complex immune microenvironment of these tumors, necessitating further exploration of biomarkers and novel combination strategies.

#### 4.2.3. New Combination Strategies, Bi-Specific Antibodies, and Antibody–Drug Conjugates

Immune checkpoint inhibitors are increasingly combined with tumor microenvironment-modulating agents to enhance efficacy in HNSCC. Angiogenesis inhibitors, such as lenvatinib, cabozantinib, zanzalintinib, and ramucirumab, are among the key drugs explored for their synergistic potential (Table 2). The LEAP-010 trial, a phase 3 study, was the first to evaluate pembrolizumab with or without lenvatinib as first-line therapy in PD-L 1-selected (CPS ≥ 1)R/MHNSCC patients, demonstrating a significantly improved objective response rate (ORR; 46.1% vs. 25.4%, *p* < 0.00001) and PFS ( 6.2 vs. 2.8 months, HR 0.64, *p* = 0.0001), but no OS benefit (15 vs. 17.9 months, HR 1.15) due to increased toxicity, reinforcing pembrolizumab alone or with chemotherapy as the standard of care [117]. Similarly, a phase 2 trial (NCT03468218) of pembrolizumab plus cabozantinib, a VEGFR, MET, AXL, and FLT3 tyrosine kinase inhibitor, reported a 52% ORR and median PFS of 12.8 months, driven by dual targeting of VEGF and PD-1 pathways, with a 2-year PFS of 32.6% and manageable toxicity, suggesting a promising alternative for R/M HNSCC [118]. In contrast, the ongoing STELLAR-305 trial (NCT06082167), a phase 2/3 study, is investigating pembrolizumab combined with zanzalintinib, a more selective multikinase inhibitor targeting VEGFR2, MET, AXL, and MER, aiming to improve tolerability and efficacy; results are awaited (ClinicalTrials.gov, NCT06082167). These trials highlight the potential and challenges of combining checkpoint inhibitors with angiogenesis modulators in R/M HNSCC. Eftilagimod alpha, a soluble LAG-3 protein, combined with pembrolizumab, showed a 29.7% ORR in PD-L1-negative first-line R/M HNSCC, offering a chemotherapy-free option for a high-unmet-need group [119].

Bi-specific antibodies are an emerging frontier in HNSCC therapy, with MCLA-158 (petosemtamab) demonstrating a preliminary ORR of approximately 30% inR/M HNSCC by targeting EGFR and LGR5, surpassing the 23.5% ORR in CheckMate 651 (see above) and 17–18% in EAGLE (see above) (Table 2) [114,115]. Similarly, ivonescimab, a PD-1/VEGF bi-specific antibody, combined with ligufalimab (anti-CD47), achieved a 65% ORR and 7.1 months median PFS in first-line PD-L1-positive R/M HNSCC, outperforming ivonescimab alone (40% ORR, 5.0 months PFS), suggesting enhanced efficacy through dual immune and angiogenic targeting [120]. Additionally, BCA101, a bifunctional EGFR/TGFβ inhibitor, combined with pembrolizumab, yielded a 48% ORR in first-line R/M HNSCC, particularly in HPV-negative patients (65%), with a tolerable safety profile marked by acneiform rash (Table 2) [121]. These agents, like MCLA-158 and SI-B001, leverage EGFR’s role, as seen in CheckMate 651’s EXTREME arm, while GEN1046 aligns with PD-L1 strategies in LEAP-010 and KEYNOTE-689. Unlike phase 3 trials such as KEYNOTE-689 (neoadjuvant/adjuvant) or CheckMate 651 (first-line), these bi-specific trials are early-phase (phase 1/2), reflecting a developing field. The lack of phase 3 data and small HNSCC cohorts, except for MCLA-158, warrants caution, especially compared to failed combinations like epacadostat in NCT03650764 or tremelimumab in EAGLE, highlighting both promise and uncertainty (ClinicalTrials.gov, NCT03650764). So, we are awaiting the phase III results to understand the real impact of these kinds of therapies.

Antibody–drug conjugate (ADC) combined a monoclonal antibody (mAbs) covalently attached to a cytotoxic drug via a chemical linker. The antitumor activity of ADC is currently explored in advanced HNSCC patients demonstrating promising potential in challenged patients. Enfortumab Vedotin, an ADC directed against Nectin-4, is investigated in subjects with locally advanced or metastatic malignant solid tumors, including HNSCC (EV-202, NCT04225117). The ORR was 23.9%, and the median PFS and OS were 3.94 and 5.98 months, respectively [122]. Sacituzumab govitecan (SG), a Trop-2 (Trophoblast cell-surface antigen 2)-directed ADC, is investigated in the TROPiCS–03 trial (NCT03964727), a phase II open-label multicohort study in patients with metastatic solid tumors. In the HNSCC cohort, which included locally recurrent or metastatic HNSCC patients heavily pretreated, SG monotherapy resulted in an ORR of 16% and reduced tumor volume in approximately half of study participants. The median PFS was 4.1 months, and the median OS was 9.0 months [123]. Another ADC that is evaluated in HNSCC patients is Tisotumab Vedotin (TV)—which targets tissue factor—in the innovaTV 207 study (NCT03485209). This trial is an open-label, global, phase 2, multicohort, multicenter study that evaluated TV monotherapy or in combination for advanced solid tumors. Results from the full cohort show encouraging antitumor activity, with 32.5% (95% CI, 18.6–49.1) of confirmed ORR, with one complete response and 12 partial responses. Median duration of response was 5.6 months (95% CI, 3.0-NR) and median time-to-response was 1.4 months [124].

### 4.3. CAR-T Cell Immunotherapy

Throughout the history of cancer treatment, CAR-T cell therapy has emerged as a revolutionary approach. These engineered T cells express synthetic receptors specifically designed to recognize and attack cancer cells. The process involves extracting T cells from patients, genetically modifying them to express CARs that recognize specific proteins on the surface of malignant cells and then reinfusing the modified T cells back into the patient [125].

A CAR is composed of three main regions: the extracellular domain, the transmembrane region, and the intracellular domain. Over successive generations, CARs have been refined to enhance functionality and minimize side effects. Second- and third-generation CARs incorporate co-stimulatory domains, such as CD28 or 4-1BB, to improve T cell activation and persistence. Fourth-generation CARs include cytokine release domains to further potentiate immune responses, while fifth-generation CARs feature cytoplasmic IL-2 receptor domains to enhance therapeutic efficacy [126].

In the context of HNSCC, CAR-T cell therapy aims to counteract immunosuppression induced by TME and strengthen the body’s natural immune response against malignant cells. Preclinical studies and early-phase clinical trials have shown promising results in certain patient populations, with CAR-T cells demonstrating potent antitumor activity and improved OS. However, key challenges remain elusive, including the selection of optimal target antigens, management of therapy-related toxicity, and ensuring long-term T cell activity. Different target antigens are subject of investigation for CAR-T cell, such as ErbB family, MUC1, CD70, and CD98hc.

#### 4.3.1. ErbB Family

The ErbB family of receptors comprising EGFR, HER2, ErbB3, and ErbB4 plays a crucial role in cell growth, survival, and differentiation. Since these receptors are frequently overexpressed in several cancers, including HNSCC, they have emerged as prime targets for CAR-T cell immunotherapy [127]. HER2, a key member of the ErbB family, regulates cell proliferation and differentiation. While HER2 overexpression is well documented in breast and ovarian cancers, it is also a potential target for CAR-T therapy in HNSCC [128]. Its role in tumorigenesis, mediated through aberrant signaling pathways, makes it a promising candidate for immunotherapy.

ErbB3, another ErbB family member, lacks intrinsic kinase activity but forms heterodimers with EGFR and HER2, activating downstream signaling pathways involved in carcinogenesis. In HNSCC, ErbB3 overexpression predicts a poor clinical outcome [129]. Targeting ErbB3 with CAR-T cells could disrupt these oncogenic signals and suppress tumor growth. ErbB4, though less commonly targeted in clinical oncology compared to EGFR and HER2, regulates cell differentiation and survival. Its potential as a therapeutic target in HNSCC remains under investigation.

EGFR has long been a well-established target in HNSCC therapy due to its frequent overexpression in tumors, where it drives cancer cell proliferation, survival, and invasion. CAR-T cell strategies targeting EGFR aim to inhibit its signaling pathways, thereby reducing tumor growth and metastasis.

A recent T4 immunotherapy trial assessed the safety and efficacy of ErbB-targeted CAR-T cells in patients with recurrent, treatment-refractory HNSCC [130]. The study specifically targeted tumors overexpressing one or more ErbB family members (EGFR, HER2, ErbB3). Results indicated that T4 CAR-T therapy was safe and did not cause significant off-tumor toxicity. However, while safety outcomes were promising, not all patients exhibited sustained tumor responses, underscoring the need for further optimization.

#### 4.3.2. MUC1

MUC1 is a mucin protein that plays a critical role in maintaining epithelial integrity. It is frequently overexpressed in cancers such as breast, ovarian, and colorectal malignancies, contributing to tumor progression through its involvement in cellular signaling and adhesion. A study by Mei Zi et al. explored the efficacy of second- and fourth-generation CAR-T cells targeting MUC1 [131]. The fourth-generation CAR-T cells, engineered to secrete IL-22, demonstrated superior cytotoxicity against MUC1-expressing HNSCC cells in both in vitro and in vivo models. In NOD/SCID mouse models, treatment with CAR-MUC1-IL22 T cells significantly reduced tumor growth and increased CD3+ lymphocyte infiltration. These findings provide strong preclinical evidence supporting MUC1-targeted CAR-T therapy for HNSCC, highlighting its potential for clinical application.

#### 4.3.3. CD70

CD70, also known as CD27 ligand (TNFSF7), is a member of the tumor necrosis factor ligand superfamily. It is transiently expressed on activated B cells, T cells, NK cells, and mature DCs, playing a pivotal role in immune activation via its interaction with CD27. Under normal physiological conditions, CD70 expression is tightly regulated. However, its aberrant overexpression has been observed in various cancers, including Hodgkin and non-Hodgkin lymphomas, as well as certain solid tumors. Persistent CD70 expression promotes tumor growth, inhibits apoptosis, and facilitates immune evasion [132].

In HNSCC, CD70 is overexpressed in certain subtypes, particularly in tumors of the larynx, oral cavity, and tongue, but not in those arising from the hard palate, hypopharynx, lip, or oropharynx. Preclinical research has validated CD70 as a promising target for CAR-T therapy, demonstrating its selective expression on cancer cells with minimal off-target effects. However, variability in CD70 expression among patients necessitates further optimization, particularly in the context of personalized medicine approaches [133].

#### 4.3.4. CD98hc

CD98hc (SLC3A2) is a transmembrane protein involved in integrin signaling, cell adhesion, migration, and tumorigenesis [134]. It has been identified as a key marker of radioresistance in HNSCC, making it a potential target for combined radiotherapy and immunotherapy [135].

Studies suggest that low expression of immune-related genes correlates with poorer prognosis in HNSCC patients, while high CD98hc expression is associated with increased resistance to radiation [136]. Given its role in tumor resilience, CD98hc has emerged as a viable candidate for CAR-T cell therapy. Development of CD98hc-targeted UniCAR-T cells has shown promising antitumor activity in three-dimensional HNSCC tumor spheroid models [137]. Moreover, combining fractionated radiotherapy with CD98hc-directed UniCAR-T therapy has demonstrated synergistic effects, effectively overcoming radiation resistance. This combinatorial approach holds significant potential for improving clinical outcomes in HNSCC.

While CAR-T cell therapy offers new hope for patients with refractory HNSCC, several challenges remain elusive. Tumor-induced immunosuppression, characterized by the presence of regulatory immune cells and inhibitory cytokines, can impair CAR-T cell efficacy. Additionally, the dense extracellular matrix of solid tumors creates a physical barrier that restricts CAR-T cell infiltration. Target antigen selection is another critical hurdle. Unlike hematologic malignancies, solid tumors lack unique tumor-specific antigens, increasing the risk of off-target effects. Furthermore, although CAR-T therapies have demonstrated high response rates, maintaining durable antitumor activity remains an ongoing challenge [138]. It also carries the risk of severe side effects such as cytokine release syndrome (CRS) and immune effector cell-associated neurotoxicity syndrome (ICANS) [139]. Despite these obstacles, ongoing progress in CAR-T cell engineering is paving the way for more efficient and sustainable immunotherapeutic approaches in HNSCC.

### 4.4. Targeting Epigenetic Alteration in HNSCC

Epigenetic alterations refer to changes in gene expression that do not involve modifications to the underlying DNA sequence. These changes can play a significant role in the progression of HNSCC by influencing cellular processes such as differentiation, proliferation, and apoptosis. Targeting epigenetic modifications has emerged as a promising therapeutic strategy for HNSCC. Several agents and drugs, including DNA methyltransferase inhibitors (DNMTis) and histone deacetylase inhibitors (HDACis), are being investigated for their potential to reverse aberrant epigenetic changes and restore normal gene function [140]. Notably, epigenetic alterations in HNSCC not only influence tumor cell behavior but also impact the TME and are closely related to immune evasion and immune response.

Aberrant DNA methylation can alter the recruitment and activation of immune cells by the expression of cytokines and chemokines, which affect the ability of T cells and DCs, and the macrophage polarization. Furthermore, histone modifications can influence the expression of MHC-I reducing the ability of CD8+ T cells to recognize tumor antigens [141,142]. In addition, epigenetic changes can regulate the expression of PD-L1, which is often upregulated in HNSCC. These checkpoints inhibit T cell activity and promote immune evasion [143]. Then, DNA methylation, histone modifications, and non-coding RNA expression can either enhance or suppress the expression of these immune checkpoint genes, affecting the ability of the immune system to attack tumor cells effectively and play a pivotal role in the development and progression of HNSCC. Now, targeting epigenetic alterations could be a possibility to restore immune surveillance.

#### 4.4.1. DNA Methyltransferase Inhibitors (DNMTis)

DNA methylation involves the addition of a methyl group to the cytosine base in DNA, typically leading to gene silencing. In HNSCC, the hypermethylation of tumor suppressor genes is common and contributes to tumorigenesis. Importantly, a report from Yang et al. analyzing human OSCC tissue microarrays showed that the high expression level of DNA methyltransferase-1 (DNMT1) was associated with the immunosuppressive molecules VISTA and PD-L1 and with a poor prognosis [144]. Moreover, DNMT1 inhibition induces the reduction in MDSCs, leading to tumor growth in an experimental model of OSCC. In a more recent work by Liu et al., the shRNA-specific DNMT1 knockdown caused an extensive DNA hypomethylation in tumor cells inhibiting the activation of proliferative pathway PI3K-AKT [145]. Likewise, some agents such as 5-azacitidine and decitabine (5-aza-2′-deoxycytidine) (DNMTis) can reverse aberrant DNA methylation patterns, leading to the reactivation of tumor suppressor genes and inhibiting cancer cell growth [143]. In particular, the effect of decitabine, currently approved for the treatment of myeloid malignancies, was studied on circulating mononuclear cells of OSCC patients and compared to healthy donors. The expression of PD-1, CTLA-4, LAG3, TIM-3, VISTA, and TIGIT, among others, was different after decitabine treatment, for instance, the expression of TIM-3 was significantly higher in CD8+ T cells of OSCC patients, while the treatment with the DNMTi decitabine enhances the expression of co-stimulatory molecules that promote the activation of effector immune cells such as OX40.

In a study by Calanca et al., the expression levels of genes related to immune markers and methylated in long coding RiboNucleic Acids (lncRNAs) were investigated using the data of OSCC patients from TCGA [146]. Here, the hypomethylation of three immune-associated lncRNAs (MEG3, MIR155HG, and WFDC21P), together with the upregulation of immunosuppressive factors such as granzime B, IL-10, and TGF β, and hypoxia inducible factor-1 alpha (HIF1A), among others, was detected, thus indicating that DNA methylation could be involved in the development of a suppressive TME.

#### 4.4.2. Histone Methyltransferase and Deacetylase Inhibitors

The histone methyltransferase KMT2D catalyzes methylation of histone H3K4 inducing the opening of chromatin and the activation of target genes; in addition, it represents the most frequently altered epigenetic modifier in HNSCC [142]. It has been observed that KMT2D directly targeted the chemokine CCL2 gene, which is related to promoting a M2 profile on TAMs and then an immunosuppressive TME. In addition, the Enhancer of Zeste 2 Polycomb Repressive Complex 2 Subunit (EZH2) is responsible for the tri-methylation of lysine 27 on histone H3 (H3K27me3). In a recent study by Zhou et al. a negative correlation was observed between the expression levels of EZH2 and class I MHC in a HNSCC cohort from TCGA. At the same time, the authors demonstrated that both pharmacologic inhibition and genetically reduced EZH2 expression resulted in a better antigen presentation by inducing a high MHC-I expression in a preclinical model of HNSCC [147]. Additionally, they observed an enhanced tumor specific T-cell proliferation and cytokine production.

Furthermore, histone deacetylation results in a closed chromatin structure and transcriptional repression. HDACis, like vorinostat and romidepsin, act by preventing histone deacetylation, leading to a more open chromatin configuration and enhanced gene transcription. This can reactivate silenced tumor suppressor genes and induce cancer cell death.

Possibly combining epigenetic therapies with ICIs could offer a synergistic approach to overcoming resistance mechanisms and enhancing antitumor responses in HNSCC. Clinical trials are currently exploring these combinations to determine their efficacy and safety in HNSCC patients. For instance, a phase II trial combining the anti PD-1 antibody pembrolizumab with the HDACi vorinostat in R/M HNSCCs and salivary gland cancers showed a promising ORR [148]. A second ongoing clinical trial is evaluating safety and outcome of the combination of anti PD-L1 durvalumab plus anti CTLA-4 tremelimumab with the DNMTi azacitidine in patients with R/M HNSCCs who have progressed on ICIs monotherapy (NCT03019003).

Then, targeting epigenetic alterations represents a frontier in HNSCC treatment, providing hope for improved therapeutic outcomes and long-term survival for patients with this challenging disease.

## 5. Conclusions

HNSCC is a complex and heterogeneous disease that remains a significant global health concern. The success of cancer immunotherapy has demonstrated that immune cells can eliminate tumor cells. Despite sustained clinical efficacy, only a fraction of cancer patients benefits from these treatments. A crucial part of combating tumors involves the interaction between immune cells, which can either support or inhibit tumor growth, and the TME, creating a delicate balance inside them. Here, we have described several mechanisms to understand how tumors evade immune system attacks. HNSCC escapes immune surveillance and resists both conventional and novel treatments by losing HLA expression, producing cytokines and factors that suppress the immune response, attracting suppressor cells, and increasing immune checkpoint molecules.

The approval of ICIs for metastatic or recurrent HNSCC marked a transformative change, offering improved survival outcomes for a group of patients. However, the variability in response rates underscores the need for further research into predictive biomarkers and combination strategies. In this context, emerging therapies, such as CAR T-cell therapy, bi-specific antibodies, antibody–drug conjugates, and epigenetic modulators show potential for overcoming immunosuppression within tumor tissues and restoring the antitumor capability of immune cells. Preclinical and early clinical studies suggest that targeting specific antigens (e.g., ErbB family, MUC1, CD70, CD98hc) and reversing epigenetic silencing could enhance antitumor immunity. However, some challenges remain related to toxicity, antigen selection, and achieving sustained responses.

## Figures and Tables

**Table 1:** Immune checkpoint inhibitors targeting the PD-1/PD-L1 and CTLA-4 in HNSCC.

Drugs	Target	Type	Fc Engineering	Half-Life (Days)	ADCC/ADCP Activity	Unique Mechanism/Features	Key Trials in HNSCC	Approval in HNSCC
Pembrolizumab	PD-1	Humanized IgG4κ	Hinge-stabilized (S228P) to prevent Fab-arm exchange	~26	no	Targets PD-1; strong correlation with PD-L1 CPS ≥ 20 for efficacy	KEYNOTE-048, KEYNOTE-040	FDA-approved: 1L (CPS ≥ 1) or 2L+
Nivolumab	PD-1	Human IgG4κ	Hinge-stabilized (S228P)	~25	no	Binds PD-1 with higher affinity than pembrolizumab; no PD-L1 CPS requirement for 2L+ approval	CheckMate 141	FDA-approved: 2L+ (post-platinum)
Atezolizumab	PDL-1	Humanized IgG1κ	Fc null (N298A mutation) to eliminate effector function	~27	no	Blocks PD-L1 binding to both PD-1 and B7-1 (CD80); may enhance T-cell priming	IMvoke, CITYSCAPE	Investigational
Avelumab	PD-L1	Human IgG1λ	Wild-type Fc	~6	yes	Retains ADCC activity; may kill PD-L1⁺ Tregs or tumor cells	JAVELIN Head & Neck 100	Investigational
Durvalumab	PD-L1	Human IgG1κ	Fc silenced (L234F, L235E, P331S)	~18	no	Engineered to minimize FcγR binding; often paired with tremelimumab (CTLA-4) in combos	HAWK, KESTREL	Investigational
Ipilimumab	CTLA-4	Human IgG1κ	Wild-type Fc	~15	Yes (theoretical)	Targets CTLA-4 on T-cells; depletes Tregs via ADCC in TME	CheckMate 651 (combo with nivo)	Not approved (investigational)
Trememilumab	CTLA-4	Human IgG2λ	No Fc effector function (IgG2 subclass)	~22	no	IgG2 limits Fc-mediated effects; focuses on CTLA-4 blockade without T-cell depletion	KESTREL, EAGLE	Investigational

**Table 2:** New combination strategies and bi-specific antibodies.

Drugs	Target	Trial-Phase	Key Trial Results
Lenvatinib + Pembrolizumab	VEGFR1-3, FGFR	LEAP-010—Phase 3	ORR: 46% (1L CPS ≥ 1); PFS: 6.3 mo; no OS benefit.
Cabozantinib + Pembrolizumab	VEGFR 1-3, MET, KIT, AXL, FLT3	Phase 2	ORR 52%, PFS 12.8 mo
Zanzalitinib (XL092) + Pembrolizumab	MET, VEGFR2, AXL, MER	STELLAR 305—Phase 2/3	Trial ongoing
Eftilagimod Alpha (IMP321) + Pembrolizumab	LAG-3 (MHC II agonist)	TACTI-002 Part C—Phase 2	ORR: 30%; PFS 2.3 mo; 12-mo OS rate 46%.
Petosemtamab (MCLA-158) + Pembrolizumab	EGFR × LGR5 bispecific	Phase 2	ORR: 30% in EGFR-resistant HNSCC
Ivonescimab (AK112)	PD-1 × VEGF-A bispecific	Phase 2	ORR: 40%; PFS 5 mo
Ligufalimab (AK117)	CD47 (SIRPα inhibitor)	Phase 2	Combined with Ivonescimab ORR: 65%. PFS 7.1 mo
BCA101	EGFR × TGF-β bispecific	Phase 1	ORR: 48% in EGFR + HNSCC
SI-B001	EGFR × PD-L1 bispecific	Phase 1/2	ORR: 30%
Enfortumab Vedotin	Nectin-4 (ADC)	Phase 2	ORR 23.9%, PFS 3.94 mo, OS 5.98 mo
Sacituzumab Govitecan	Trop-2 (ADC)	Phase 2	ORR 16%, PFS 4.1 mo, OS 9 mo
Tisotumab vedotin	Tissue factor (ADC)	Phase 2	ORR 32.5%

## Abbreviations

The following abbreviations are used in this manuscript:

5-FU	5-fluorouracil
ADC	Antibody–drug conjugate
ADCCJ	Antibody-dependent cell-mediated cytotoxicity
ARG1	Arginase 1
B2M	Beta 2 microglobulin
Breg	Regulatory B cell
CAF	Cancer-associated fibroblast
CAR	Chimeric antigen receptor
CDC	Complement-dependent cytotoxicity
CIITA	Class II major histocompatibility complex transactivator
CPS	Combined positive score
CRT	Cisplatin-based chemotherapy
CTL	Cytotoxic T lymphocyte
CTLA-4	Cytotoxic T lymphocyte antigen 4
CXCL	C-X-C motif chemokine ligand
DC	Dendritic cell
DNMT1	DNA methyltransferase-1
DNMTis	DNA methyltransferase inhibitors
EC	Endothelial cells
ECM	Extracellular matrix
EFS	Event-free survival
EGFR	Epidermal growth factor receptor
FDA	Food and Drug Administration
GM-CSF	Granulocyte/monocyte-colony stimulating factor
HDACis	Histone deacetylase inhibitors
LOH	Loss of heterozygosity
HLA	Human leukocyte antigen
HNSCC	Head and neck squamous cell carcinoma
HPV	Human papilloma virus
ICP	Immune checkpoint
ICPi	Immune checkpoint inhibitors
IDO	Indolamine-2,3-dioxygenase
IFN	Interferon
IgG	Immunoglobulin
IL	Interleukin
LAG-3	Lymphocyte-activation gene 3
LAHNC	Locally advanced head and neck carcinoma
LncRNAs	Long coding ribonucleic acids
LRF	Reducing locoregional failure
LRT	Locoregional treatment
LSCC	Laryngeal squamous cell carcinoma
MDSC	Myeloid-derived suppressor cell
MHC	Major histocompatibility complex
NETs	Neutrophil extracellular traps
NK	Natural killer
NLR	Neutrophil-to lymphocyte ratios
NO	Nitric oxide
OPSCC	Oropharyngeal squamous cell carcinoma
ORR	Overall response rate
OS	Overall survival
PD-1	Programmed death receptor 1
PD-L1	Programmed death ligand 1
PFS	Progression-free survival
PGE2	Prostaglandin E2
PI3KPMN	Phosphatidyl-linositol-3-kinasePre metastatic niches
PTEN	Phosphatase and tensin homolog
R/M	recurrence and or metastatic
RNS	Reactive nitrogen species
ROS	Reactive oxygen species
RT	Radiotherapy
TAMs	Tumor-associated macrophages
TANs	Tumor-associated neutrophils
TCGA	The Cancer Genome Atlas
TCR	T cell receptor
TGFBR2	Transforming growth factor beta
TGFβ	Transforming growth factor beta
TIGIT	T cell immunoglobulin and ITIM domain
TIL	Tumor-infiltrating lymphocyte
TIM-3	T cell immunoglobulin and mucin domain-containing protein 3
TME	Tumor microenvironment
TNFα	Tumor necrosis factor alpha
TP53	Tumor Protein P53
Treg	Regulatory T cell
VEGF	Vascular endothelial growth factor
VISTA	V-domain Ig suppressor of T cell activation
